# Supplementary material for: Flipping the switch on some of the slowest mutating genomes: Direct measurements of plant mitochondrial and plastid mutation rates in msh1 mutants
Source: PLoS Genet. 2025 Jun 30;21(6):e1011764. doi: 10.1371/journal.pgen.1011764 (PMC12225983; doi:10.1371/journal.pgen.1011764)
Supplement: S1 Table — All tested sequences were confirmed as germline variants. (PDF) [file pgen.1011764.s001.pdf]

Table S1. Primers used to validate a sample of mitochondrial and plastid SNVs by PCR amplification and Sanger sequencing. All tested sequences were confirmed as germline variants.

Organelle	MA Line	Position	SNV	Location	F primer (5'→3')	R primer (5'→3')	Marker[1]
Mito	M1_6_F8	21077	C>T	Intron (Rps3)	CTGTTAGCGTAGCTATGCAGTGG	ACTGGTACTTGATTGGGGCG	271
	M3_2_F8	95003	T>C	CDS (Nad2)	CCTGCGTATGAGAACATAGTAATGG	CTTTGACTCTATATATGTGGGCACC	273
	M2_2_F8	191380	C>T	CDS (CcmFC)	CTCGCTTCTTCACAAGCTTACC	TATAGCCACTTCAGCCGTGC	274
	M3_8_F8	123820	A>T	intergenic	AAAGCAAGAAACCCATCCAGC	CAAGTCCTTTTAGGCGGTCC	276
	M3_5_F8	47247	A>G	intergenic	CGCTACACCGCCCTATTGAT	TGTTAGCAGGATCGGGTAGG	278
Plastid	M3_4_F8	67994	A>G	CDS (Rps18)	CAAATTGAGTGCTTGTATGTCAAC	CGAGCTTGTTTTATAGCAATAGTGA	261
	M2_8_F8	61752	T>C	CDS (PetA)	TACCTTATACATCATAACCAAATCAAAGTCG	CTCCGGTAAAATAAGAACAGCTCC	263
	M2_6_F8	73081	T>C	CDS (PsbB)	GCATTTCATGTAACAGGATTATATGGTCC	TCAGCCAGTCCAGCACTAAC	265
	M2_8_F8	6417	C>T	intergenic	CAAGTCGTACGTTGCTTTCTACC	TTCCGTCCCAGAGTACAGTC	267
	M2_5_F8	71585	A>G	Intron (ClpP)	GATTTGCACATATAGGACAAATGAACC	TCTCCCTCGATCGAGATATCC	269

[1]Marker refers to the trace number in the ab1 files for mutant, wild type, and F9 mutant progeny plants (Sanger data available via GitHub: https://github.com/dbsloan/msh1_MA_lines).
