# Supplementary material for: Flipping the switch on some of the slowest mutating genomes: Direct measurements of plant mitochondrial and plastid mutation rates in msh1 mutants
Source: PLoS Genet. 2025 Jun 30;21(6):e1011764. doi: 10.1371/journal.pgen.1011764 (PMC12225983; doi:10.1371/journal.pgen.1011764)
Supplement: S1 Fig — Each point represents the measured germination percentage for a line, typically based on a sample of 18 seeds. Additional sets of 18 were sown in cases of extremely low germination values from the first set. (PDF) [file pgen.1011764.s002.pdf]

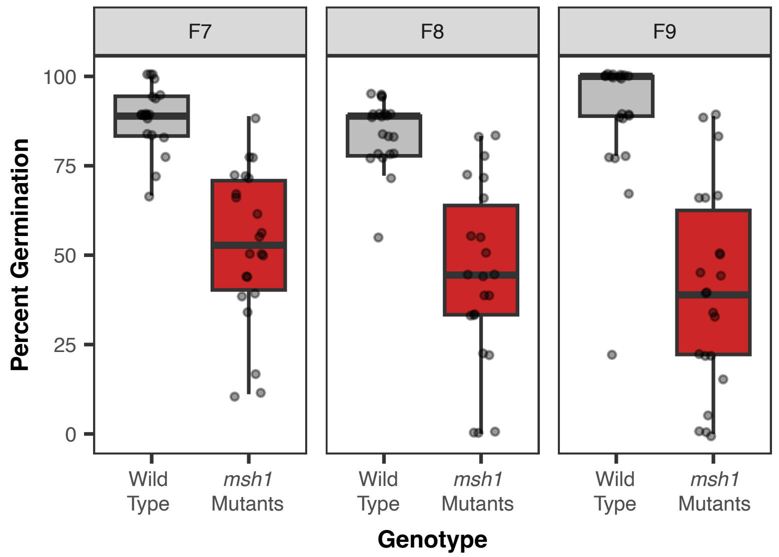

Figure S1. Reduced germination percentage in *msh1* mutant lines compared to WT lines in the F7, F8, and F9 generations (germination percentage was not tracked in previous generations). Each point represents the measured germination percentage for a line, typically based on a sample of 18 seeds. Additional sets of 18 were sown in cases of extremely low germination values from the first set.
