# Supplementary material for: Flipping the switch on some of the slowest mutating genomes: Direct measurements of plant mitochondrial and plastid mutation rates in msh1 mutants
Source: PLoS Genet. 2025 Jun 30;21(6):e1011764. doi: 10.1371/journal.pgen.1011764 (PMC12225983; doi:10.1371/journal.pgen.1011764)
Supplement: S2 Fig — Each point represents the read mapping percentage to the mitochondrial (left) or plastid (right) genome for an individual line. (PDF) [file pgen.1011764.s003.pdf]

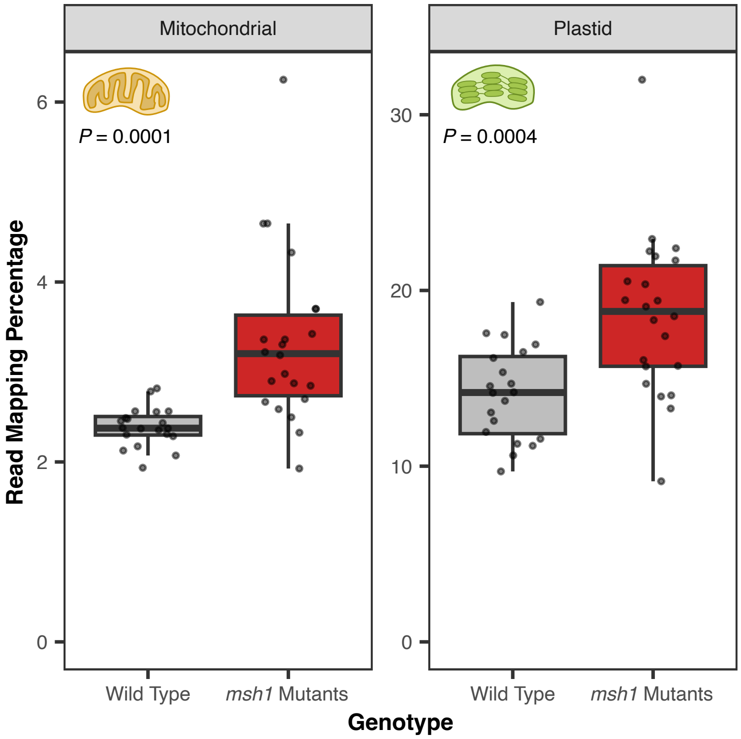

Figure S2. Total DNA from leaf tissue exhibited a higher percentage of reads mapping to organelle genomes for *msh1* mutants than WT lines. Each point represents the read mapping percentage to the mitochondrial (left) or plastid (right) genome for an individual line.
